# Impact of a Terrorist Attack on the Mental Health of Directly Exposed French Adolescents: Study Protocol for the First Step of the AVAL Cohort Study

**DOI:** 10.3389/fpsyt.2019.00744

**Published:** 2019-10-25

**Authors:** Marion Grenon, Maëlys Consigny, Christophe Lemey, Jean-Pierre Simson, Nathalie Coulon

**Affiliations:** ^1^Department of Psychiatry, Centre Hospitalier Régional Universitaire de Brest, France; ^2^INSERM CIC 1412, Centre Hospitalier Régional Universitaire de Brest, France; ^3^Department of Psychiatry, Hôpital d’Instruction des Armées Clermont-Tonnerre, Brest, France; ^4^Department of Child and Adolescent Psychiatry, Etablissement Public de Santé Erasme 92, Antony, France

**Keywords:** PTSD, terrorism, teenagers, post-traumatic stress disorders, adolescent psychiatry, crisis intervention

## Abstract

**Background:** Several terrorist attacks have recently taken place in France and Europe. Various studies have shown a high prevalence of Post-Traumatic Stress Disorder (PTSD) and other psychiatric disorders among the victims of these attacks. Nevertheless, research in this field is scarce and no cohort study has been conducted yet to evaluate the impact of a terrorist attack on teenagers directly exposed to this type of events. Therefore, we decided to work on the AVAL (*Adolescents Victimes de l’Attentat de Londres*) cohort study in order to measure the psycho-traumatic impact of this attack and to describe these adolescents’ health care pathways.

**Material and method:** The 53 students of a French high school who were directly exposed (criterion A1 of PTSD in DSM-5) to the terrorist attack perpetrated in London on March 22, 2017 constitute the target population of this monocentric cross-sectional observational study. We decided not to include the three students who were physically wounded and, therefore, didn’t have the same sensorial exposition. The primary endpoint will be the prevalence of PTSD 12 to 15 months after the attack, measured by the PCL-5 (Post-traumatic stress disorder Check-List for DSM-5) global severity score: the diagnosis of PTSD will be retained when the score is > 32. We will also use an extensive battery of clinical tests to assess the prevalence of anxiety disorders, mood disorders, sleep disorders, addictions, suicide risk, and alterations in social, family, and school functioning 12 to 15 months after the attack. We will also describe these adolescents’ health care pathways since the attack and collect data from the clinical evaluation performed during the initial intervention of the medico-psychological emergency cell within 10 days after the attack.

**Discussion:** The findings of this study are intended to provide epidemiological data about the psycho-traumatic impact of a terrorist attack on the mental health of directly exposed adolescents and to describe these adolescents’ health care pathways, thus contributing to improve the immediate, post-immediate, and delayed response strategies after a major psycho-traumatic event involving adolescents (and in particular after terrorist attacks), as well as the identification and psychiatric care of the young survivors requiring specialized care.

**Clinical Trial Registration:** www.ClinicalTrials.gov, identifier NCT03493243.

**Ethics and Dissemination:** The regional ethics committee (Comité de Protection des Personnes Ouest IV—Nantes) approved the study protocol (Reference 10/18_3). All participants (and their legal guardians, for minors) must sign the informed consent to participate. The protocol was presented at the French congress of psychiatry in Nantes (France) in November 2018. After study completion, the results will be published and detailed in Marion Grenon’s MD thesis in psychiatry.

## Introduction

Since 2012, several terrorist attacks have taken place in France and Europe. On March 22, 2017, one of these attacks, which occurred in one of the most popular tourist areas of London, stroke French high school students among other people. Around 14:40 that day, a car turned into Westminster Bridge in London. The vehicle drove on the sidewalk, running down twenty pedestrians in its path. The assailant ended his run just after Big Ben, crashing his car in the North gate of Westminster Palace, seat of the British Parliament. Armed with a knife, the driver left his vehicle and ran to the main entrance of the Parliament, then stabbed a guard and entered the courtyard of the Palace before being shot by two officers. Six people died in this attack, including the perpetrator. Fifty people were injured, including three French high school students who were on a school trip.

Apart from the three physically injured teenagers, all the students were repatriated to France the day after the attack, after being heard by the British police. Upon arrival, the medico-psychological emergency cell was deployed to allow them, their families, and anyone who felt the need to do so to meet a mental health professional (nurse, psychologist, or psychiatrist). This post-immediate evaluation involved almost all the high school students who constitute our target population and allowed us, as we will explain later, to retrospectively get a basis for our research work through their medical files.

Based on this post-immediate evaluation, the adolescents who needed it were referred to specialized care, mainly to the nearest outpatient consultation center (*Centre Médico-Psychologique*). However, it seems important to note that during the year following the attack, no specific follow-up measures were taken for these young people. The participants did not benefit from any systematic individual follow-up or group therapy, and the decision to seek or not to seek mental health care was left to each of them and their families. In addition, no commemoration was organized within or outside the school on the first anniversary of the traumatic event.

### Prevalence of Post-Traumatic Stress Disorder

For most children and adolescents, the psychological stress that occurs after a traumatic event is usually of short duration. However, for some young people, symptoms do not improve spontaneously and become clinically significant, persistent, and disabling (1).

The prevalence of post-traumatic stress disorder (PTSD) among children and adolescents who have survived disasters varies greatly from 1% to 60%, depending on the target population and the measures used to establish the diagnosis (2). One of the elements which could explain a particularly important prevalence of PTSD in this young population is the fact that acts of terrorism, unlike other types of disasters, combine a human origin, an intentional aspect, a low predictability, and a short duration (3). Indeed, unlike natural disasters, terrorist attacks are man-made disasters which carry a deliberate intention to harm and destroy (4).

Among young people exposed to terrorist attacks, previous research found increased rates of mental disorders for isolated attacks occurring in countries that were not at war (e.g., Oklahoma City bombing, September 11^th^ 2001) as well as attacks occurring in areas of continued political conflict (e.g., Guatemala, Northern Ireland, Israel) (3). In addition, according to a meta-analysis of all longitudinal studies conducted on populations directly exposed to traumatic events between 1998 and 2010, the prevalence of PTSD would increase over time after “intentional” traumatic events (attacks, war) whereas it would tend to decrease in the long term after “unintentional” traumatic events (5).

In Europe, a recent study about the post-traumatic responses to the July 22, 2011 Oslo terror among Norwegian high school students analyzed PTSD symptoms and showed that 0.8% of respondents reported substantial distress on the reexperiencing item, 4.9% on the avoidance item, and 1.1% on the hyperarousal item. Moreover, 4.9% reported substantial distress in one PTSD symptom area at least, whereas 0.4% reported substantial distress in all three areas (6). The results also revealed a gradual relationship between proximity to terrorist attacks and distress related to PTSD symptoms: the greater the personal proximity to the scene of attacks, the higher the level of distress.

### Psychiatric Disorders Following Traumatic Exposure

On the other hand, although PTSD appears to be the most commonly studied reaction, other psychological disorders can also occur following a traumatic event:

- Depressive disorders (3, 7–10);
- Emotional numbness, which would be more suggestive of a depressive response (11);
- Suicidal ideation (12);
- Anxiety disorders (7, 10);
- Behavioral disorders (7), with potential academic and non-academic consequences (13, 14);
- Sleep disorders (12).

The prevalence of anxiety disorders (e.g., social anxiety, agoraphobia) might even be higher than the one of PTSD among youth proximally exposed to terrorist attacks (15). Adolescents are also more likely to manage their symptoms by using substance abuse. Indeed, studies made after terrorist attacks in Israel and after September 11^th^, 2001 documented an increased use of alcohol, illicit substances, and nicotine among adolescents (13, 14, 16).

### Risk Factors of Post-Traumatic Stress Disorder

Several risk (and protective) factors of post-traumatic stress disorder have been identified. They can be classified in three categories according to their temporality:

- **Pre-traumatic factors**: personal or family history of mental disorders, low level of intelligence, low socio-economic level, genetic factors, gender (females), history of abuse or trauma, low pre-existing self-esteem (7, 17–23);
- **Peri-traumatic factors**: which are related to the traumatic exposure: gravity and proximity to the event, duration of exposure, but also factors related to the subjective experience of the event such as peri-traumatic dissociation and distress or the perceived threat of death (7, 17, 24–26);
- **Post-traumatic factors**: related to the subject himself (psychiatric comorbidities, ineffective coping strategies, acute stress disorder) and his environment (low social level, social isolation, poor family functioning…) (7, 24).

For children and adolescents, post-traumatic factors have been identified as being more strongly correlated with the risk of PTSD than pre-traumatic factors (7). Nevertheless, the most important predictors of PTSD seem to be peri-traumatic factors such as peri-traumatic dissociation and the perceived death threat (17). On the other hand, social support appears to be one of the most important protective factors (17, 24, 27– 29).

### The Need for Further Research

Victims of a terrorist attack, as well as those who witness or hear about these attacks, are likely to experiment long-term negative effects (30). In order to limit the devastating impact of these events, which are by definition unpredictable and can be highly traumatic, it seems essential to react as quickly and as effectively as possible. The psychological and psychiatric care of the victims of terrorist attacks therefore constitutes a major public health issue.

In addition to the potential somatic and social consequences of such events, many studies documented the impact of terrorist attacks on children’s and adolescents’ mental health and revealed a significant prevalence of PTSD, but also other psychiatric symptoms (such as anxiety and depression) among these populations (3). However, there is growing evidence of a wide range in prognosis, in terms of mental health, after an exposition to a terrorist attack: most youth seem to recover without any psychological treatment, while others experience persistent PTSD or delayed stress response (31).

Young survivors of terrorist attacks are particularly vulnerable. Indeed, post-traumatic stress is likely to compromise their psycho-social development and academic success with potential long-term effects (32). Therefore, they may need long-term health monitoring in terms of primary and secondary care (33). Nevertheless, several studies showed that in the field of mental health, many needs are not being met in the aftermath of terrorist attacks (34, 35). In addition, existing research in this area is scarce and essentially includes cross-sectional studies (36, 37).

To our knowledge, only one cohort study has been conducted in Europe to assess the impact of a terrorist attack on the mental health of directly exposed adolescents (10). As these attacks frequently involve children and adolescents (37–39), further research is needed to evaluate the use of the different types of existing care structures for young survivors as well as their long-term needs. Therefore, we decided to set up this cross-sectional observational study which is the first step in the constitution of the AVAL cohort study, a cohort of French high school students who were directly exposed to the terrorist attack that took place on Westminster Bridge (London, England) on March 22, 2017.

## Aims

The primary aim of this study is to evaluate the psycho-traumatic impact of a terrorist attack on the mental health of directly exposed adolescents 12 to 15 months after the attack, measuring the prevalence of post-traumatic stress disorder, anxiety disorders, mood disorders, sleep disorders, addictions, and suicide risk in the target population.

Our secondary aims are clinical, epidemiological, preventive, and therapeutic. We will indeed search for alterations in social, family, and school functioning (clinical aim), but also tend to identify which factors are associated with the risk of developing PTSD, anxiety disorders, or depressive disorders 12 to 15 months after the attack (epidemiological aims). We also aim to provide a standardized psychiatric evaluation of the young survivors who constitute the target population, in order to identify and refer to specialists the ones who require psychiatric and/or psychological care (therapeutic aims). Finally, we aim to identify which factors are associated with the fact of seeking medical or psychological advice during the year after the attack, which will help us to assess the needs of directly exposed adolescents in terms of medical and psychological care after a terrorist attack (preventive aim).

## Methods and Analysis

### Design

This study, which will be the first step of the AVAL cohort study, will be a monocentric cross-sectional observational study. It will be performed in a single center, 12 to 15 months after the terrorist attack to which the target population was exposed.

The evaluation will consist of both self-assessment questionnaires and clinician-administered questionnaires. Both these evaluations will last around 1 h and will be performed on distinct days. The hetero-evaluation will be conducted by the same investigator for all participants. Nevertheless, in order to provide a safe space for all students and their families, and prevent any emotional difficulty, volunteers from the medico-psychological emergency cell (*CUMP-29 renforcée*) will be seconded to assist the investigator during all data collection.

Pre-existing personal and family psychiatric condition, family composition, psycho-traumatic history, stressful life events, characteristics of traumatic exposure, early evolution of disorders, and health care pathway since the attack will be explored retrospectively upon the participant’s statement. Psychological symptoms and psychiatric disorders will be assessed based on the current health status of participants at the time of evaluation.

### Patients’ Recruitment

#### Inclusion Criteria

The 53 students of a French high school who were directly exposed (criterion A1 of PTSD in DSM-5) to the terrorist attack perpetrated on Westminster Bridge in London on March 22, 2017 are the target population of this study.

#### Exclusion Criteria

We will exclude from this study the three students who were physically wounded during the attack and, therefore, didn’t have the same sensorial exposition. However, if they want to, these three teenagers will be offered the same standardized assessment as all participants, so that we can fulfill our aim of clinical screening and refer them to specialized care if necessary.

### Measures

#### Outcomes and Instruments

**Supplementary Figure 1** summarizes the variables that will be assessed in this study.

##### Post-Immediate Evaluation

We will use retrospective data from the clinical evaluation performed during the initial intervention of the medico-psychological emergency cell within 10 days after the attack. Indeed, for most of the 53 adolescents who meet the inclusion criteria, a standardized face-to-face evaluation was conducted. The psychiatric nurse, psychologist, or psychiatrist in charge of this assessment had to record the immediate and post-immediate symptoms of the patient, notify whether the adolescent had missed school or taken any medication since the attack, empirically quantify the psychological impact of the event (mild/moderate/severe), and notify whether a medical certificate had been established or not.

The immediate symptoms sought were anxiety, fear, sadness, psychic sideration, stupor, agitation, panic, confusion, derealization, dissociation, and automatic activity. The post-immediate symptoms sought were sleep disorders, difficulty concentrating, phobias, avoidance, hypervigilance, intrusion symptoms, and feeling of guilt.

Finally, for some of the 53 adolescents (depending on the mental health professional who led the evaluation), peritraumatic dissociation had been assessed using the Peritraumatic Dissociative Experience Questionnaire (PDEQ) (40). This self-assessed questionnaire consists of 10 items describing dissociative experiences which can occur during a traumatic event. Each item is measured using a 5-point Likert scale reflecting the degree to which the patient experienced the symptom that is described, from 1 (not at all true) to 5 (extremely true). The total score is the sum of all items. A score ≥ 15 indicates a significant dissociation. People with intense peri-traumatic dissociation are more likely to develop PTSD (7, 17, 24). This questionnaire has been validated for French-speaking individuals (41) and school-aged victims (42).

##### Clinical Assessment 1 Year After the Attack: Self-Assessment Questionnaires

###### Socio-Demographic Data

Age, sex, grade (for those in school), socio-economic level of parents or legal guardians, ethnic origin/country of origin, and family composition will be collected from each participant.

###### Life Event Checklist for DSM-5

The Life Event Checklist for DSM-5 (LEC-5) (43), which consists of a list of difficult life events, will allow us to look for a possible exposure to a traumatic event prior to the attack of March 22, 2017. This questionnaire explores 17 items. The first 16 correspond to events that could cause a PTSD or psychic distress, while the latter allows to evaluate any other extremely stressful event that would not have been explored by the first 16 items. This checklist will help us to gather information about former traumatic experiences of each participant. There is no formal rating protocol, but the patient may indicate several exposure levels for the same event (happened to me, witnessed it, learned about it, part of my job, not sure, doesn’t apply). People who experienced previous traumatic events are more likely to develop PTSD (18, 22, 23) when exposed to a new one.

###### Adolescents Life-Change Events Scale

The Adolescents Life-Change Events Scale (ALCES) (44) consists of 31 items and explores stressful life events in five domains (family, school, social life, emotional life, and health). We will use this questionnaire to explore stressful life events that may have caused a significant change in the participant’s daily life during the year before the attack (first part) and since the attack (second part).

###### Sheehan Disability Scale

Sheehan Disability Scale (45) is commonly used to measure the impact of psychiatric conditions on daily life. It explores the subject’s experience and his perception of the intensity with which events have affected three common areas of his life: studies/work, social life, and family life (46). Each of these three domains is evaluated by the patient on a scale of 0 to 10. The total score ranges from 0 (unimpaired) to 30 (highly impaired), but each of the three subscales can also be scored independently (any score ≥ reveals a significant impairment).

###### Echelle De Provisions Sociales Abrégée

The Echelle de Provisions Sociales Abrégée (EPS-10) (47) assesses the declared and perceived quality of social support received by the subject. It consists of 10 items, with 2 items for each of the 5 dimensions explored: emotional support, social integration, tangible and material help, guidance, and reassurance of worth. Each item is measured using a 4-point Likert scale reflecting the degree to which the patient agrees or disagrees with the statement from 1 (strongly disagree) to 4 (strongly agree). This questionnaire will allow us to continue to explore the participants’ vulnerability to stress by assessing how much social support they feel they get. Indeed, social support appears to be one of the main factors associated with the evolution of PTSD (17, 24, 27, 28).

###### Family and School Resources

In order to identify social resources, we added eight close-ended questions (answer = yes or no) to complement EPS-10. These questions will help us to assess the perceived role, for participants, of their parents, family, and school social environment (professors, nurse, guidance counselor, etc.). These answers will be confronted with the data collected during the medical interview (see below).

###### School Performances

To complement the Sheehan Disability Scale, we added three close-ended questions (answer = yes or no) which explore alterations of school performances that may have occurred since the attack (difficulty concentrating, better or worse grades, school absenteeism, repetition, changing fields). These answers will also be confronted with the data collected during the medical interview (see below).

###### Post-Traumatic Stress Disorder Check-List for DSM-5

The Post-traumatic stress disorder Check-List for DSM-5 (48) consists of 20 items which explore the 20 symptoms of PTSD described in the DSM-5 (49). For each symptom, the patient evaluates the intensity of the symptom during the previous month on a 5-point Likert scale from 0 (not at all) to 5 (extremely). The 20 items can be grouped into four subscales corresponding to the four sub-syndromes of PTSD: intrusion symptoms (items 1 to 5, corresponding to criterion B), avoidance symptoms (items 6 and 7, corresponding to criterion C), negative of cognition and mood (items 8 to 14, corresponding to criterion D), and hyperarousal (items 15 to 20, corresponding to criterion E).

This self-questionnaire allows to obtain a total score of severity (by adding the score obtained for each of the 20 items) or an independent severity score for each criterion from B to E. It can also be used as a diagnostic scale, which is how we will use it: the diagnosis of PTSD will be retained when the global severity score > 32 (48). The prevalence of PTSD 12 to 15 months after the attack, assessed using PCL-5, will be the primary endpoint of this study.

###### Peritraumatic Distress Inventory

The Peritraumatic Distress Inventory (PDI)(42,50) consists of 13 items and assesses the emotional distress reactions experienced during or immediately after exposure to a traumatic event. Each item is measured using a 5-point Likert scale reflecting the degree to which the patient experienced the symptom that is described, from 0 (not at all true) to 4 (extremely true). The total score is the sum of all items. A score ≥ 15 indicates significant distress. People with severe peri-traumatic distress are at higher risk of developing PTSD (50).

##### Clinical Assessment 1 Year After the Attack: Medical Interview and Clinician-Administered Questionnaires

All the questionnaires will be administered by the same clinician who is a psychiatry resident (8th year of medical school) supervised by a child and adolescent psychiatrist, and a military psychiatrist who are both trained in psycho-traumatology and have professional experience in this field.

Exceptionally, volunteers from the medico-psychological emergency cell will be seconded to assist the investigator during data collection. Two volunteers will be present each day. One of them will be present during the medical interviews in order to be able to offer the participant a space for dialogue at the end of the interview if necessary, while the second one will be on standby in the high school and welcome any person (student, family, teachers, etc.) who would feel the need to come and talk about the attack.

###### Genogram

At the beginning of the medical interview, over 15 min, the clinician will produce a genogram, which aims to represent the participant’s family on three generations and to highlight the relationships between the adolescent and each member of these three generations. During this interview, the clinician will also search for a pre-existing personal and family psychiatric condition, and ask the patient about his or her hobbies, professional project, and romantic relationships. This introductory time also aims to allow the participant to feel safe and confident in this unknown context of clinical evaluation.

###### MINI International Neuropsychiatric Interview for Children and Adolescent (M.I.N.I. Kid 7.0.2)

The MINI International Neuropsychiatric Interview (51) is a brief structured diagnostic interview developed by American and European psychiatrists and clinicians. Although it can also be used as a self-assessment questionnaire, it lowers its psychometric performances, which is why we chose to use it as a clinician-administered questionnaire.

The MINI Kid, which is dedicated to children and adolescents, explores the most frequent and most relevant disorders in child psychiatry: mood disorders, suicidality, anxiety disorders and PTSD, addictions, motor disorders, hyperactivity, behavioral disorders, eating disorders, and psychotic disorders.

David V. Sheehan, as “copyright holder” of the all versions of “MINI International Neuropsychiatric Interview for Children and Adolescents - M.I.N.I. Kid 7.0.2 (8/8/16 version),” granted permission to Nathalie Coulon, MD-PhD in CHU Brest (France), as healthcare providers, to use this interview under terms and conditions which we respected.

###### Psychoactive Substances Use

To complement the MINI Kid, we will use eight close-ended questions to explore whether the psychoactive substances use has changed or not since the attack. We will focus in particular on alcohol, tobacco, and cannabis, which are the most frequently used substances in the age group of the target population.

###### World Health Organization Disability Assessment Schedule 2.0

In order to explore any functional impairment, the World Health Organization Disability Assessment Schedule 2.0 (WHODAS 2.0), which was developed in connection with DSM-5 (49), will be used to measure health and disability through six areas of daily life: cognition, mobility, self-care, getting along/interacting with other people, life activities, and participation (joining in community activities). As a generic instrument, WHODAS 2.0 can be used to measure the functional impact of any pathology. The full version consists of 36 questions, but we chose to use the short version administered by an investigator, which brings together 12 items. These 12 questions explore the operational difficulties experienced over the last 30 days in the six areas detailed above. The 12-item short version explains 81% of the variance of the long version.

###### Psychological and Medical Care

To conclude the medical interview, the clinician will explore the psychological and medical care that the participant may have sought and/or received since the attack. Using nine questions (eight close-ended and one semi-open), we will explore the health care pathway followed by the participant since the attack, providing information about the types of medical and/or psychological care the patient received (medical/psychiatric/psychological care, psychotherapy/medication), when (during the first week following the attack or not) and where (type of healthcare structure).

If the participant did not seek any psychological or medical care, the clinician will notify why (the participant didn’t want to, the participant’s parent or legal guardians didn’t want him/her to, the participant didn’t feel like he or she needed help, the participant or his/her legal guardians didn’t know where to go to get help, the participant or his/her legal guardians thought it was too soon to get help, other reason).

Finally, to limit the clinical bias in our evaluations, we will notify any current medication.

#### Statistical Analysis

Descriptive statistics of the population will include the number of missing data, the mean, the standard deviation, the median, the minimum, and the maximum for continuous variables, as well as frequency and percentage for qualitative variables.

The baseline characteristics of the sample will be compared between adolescents who did or did not develop PTSD using comparison of means (Student test or Wilcoxon test) or of frequencies (Chi-square test or Fisher’s exact test).

Analysis of correlation will also be performed in order to investigate whether some of the factors we evaluate might play a role in the development of PTSD.

## Discussion

To our knowledge, only one cohort study has been conducted in Europe to assess the impact of a terrorist attack on the mental health of directly exposed adolescents (10). However, in that study, most interviews were conducted 5 to 6 months after the attack, therefore providing a very different timeframe. In addition, according to its authors, that study lacked “*measures of pre-trauma health conditions, demographic information and psychosocial interventions post-trauma*,” which may have influenced the outcome of the study (10). In our study, we will try to address these limitations by collecting data about exposition to previous traumatic events, family composition and relationships, pre-existing personal, and family psychiatric condition as well as the psychological and medical care that the participant may have sought and/or received since the attack.

In addition, as all the members of our target population were going to the same high school, we will be able to identify all the survivors who meet our inclusion criteria, therefore allowing us to hope for an exhaustive recruitment of the target population.

The early intervention of the medico-psychological emergency cell, who operated within 10 days after the attack, will also allow us to collect precious retrospective clinical data such as the immediate and post-immediate symptoms of the participant, early school absenteeism, and quantified post-immediate psychological impact of the event (mild/moderate/severe).

Finally, the fact that the hetero-evaluation will be conducted by the same investigator for all participants reinforces the power of our study. However, we made sure that this investigator would be accompanied, throughout the collection of data, by mental health volunteers who have been trained to take care of people facing a traumatic event, which will allow us to provide a safe space for all participants and their families.

Despite these strengths, our study is subject to several limitations. First, due to the cross-sectional design of the study, we will not be able to establish causal relationships. Second, with a target population of only 53 individuals, the number of patients will necessarily be limited. Third, the data collected through the post-immediate evaluation was gathered outside of any protocol at that time, which makes it barely scientifically exploitable. This initial data is thus very different from the one we will collect 12 to 15 months after the attack, which will not allow us to compare them.

Fourth, although the home and school environment seem to play a key role in modulating children’s and adolescents’ reaction to terrorism (7, 29, 52–54), our evaluation of this environment will be superficial. It would be interesting to conduct a real systemic assessment among these adolescents including interviews with their teachers and families, but unfortunately this cannot be done as part of this study.

Finally, it seemed to us that collecting data for this study 1 year after the terrorist attack would increase our chances of including most of the target population while minimizing the risks of disruption for the students and their families, since they would probably already be thinking about the event. However, the choice of this date could be a source of bias. Indeed, it is possible that the symptoms of post-traumatic stress, depression, and anxiety are more important around the first anniversary of the traumatic event.

Another potential source of bias could come from the fact that the teenagers who constitute our target population were particularly preserved from the mediatic exposition in the aftermath of the attack. Media participation can be perceived as distressing by terrorist attack survivors and be associated with more post-traumatic stress reactions (55). As explained by Aakvaag et al., “*Survivors may also experience that the portrayal of them as a group in media or other contexts is overly heroic or positive, which may not correspond with their private experience of trauma*” (56). However, on the other hand, the massive public attention of mass trauma such as terrorist attacks can result in surrounding populations expressing their support for and sympathy with victims (57). Therefore, it is also possible that the low coverage of this attack by French media deprived the participants of a social support that could have reduced their risk of developing post-traumatic stress reactions. Indeed, as explained earlier, social support appears to be one of the main factors associated with the evolution of PTSD (17, 24, 27, 28).

Finally, group effects could also be a source of bias in this study. Indeed, all the participants went to the same school at the time of the attack and most of them stayed in the same high school afterwards, which implies that just as it was the case with the victims of the Beslan terrorist attack, the participants had to face other victims of the traumatic event on a daily basis. On the one hand, it might have been helpful for them to get mutual support from people who share similar experiences. However, on the other one, daily confrontation with other victims of the same attack seems likely to act as a constant reminder of the traumatic event and therefore to increase the risk of developing post-traumatic stress reactions.

This study has a major preventive aspect for the target population. Indeed, all directly exposed adolescents will be offered the same standardized assessment and receive a message of prevention with contact details of caregivers, whether they want to be included in the study or not, so that we can fulfill our aim of clinical screening and refer them to specialized care if necessary.

This study is also intended to provide epidemiological data about the psycho-traumatic impact of a terrorist attack on the mental health of directly exposed adolescents and to describe these adolescents’ health care pathways. These clinical and epidemiological information could help healthcare professionals and public authorities to better adapt their immediate, post-immediate and delayed response strategies after a major psycho-traumatic event involving adolescents, and in particular after terrorist attacks. Finally, assessing healthcare pathways followed by participants since the attack will help us to evaluate the needs of directly exposed adolescents in terms of medical and psychological care after a terrorist attack and, if necessary, to alert public authorities to needs which are not being met.

## Ethics Statement

As it involves human participants, the protocol of this study was approved by Comité de Protection des Personnes Ouest IV—Nantes. Written informed consent to participate in this study was provided by the participants’ or their legal guardian for minors.

## Author Contributions

J-PS was one of the psychiatrists from the initial intervention and had the idea to develop a study. MG designed the protocol with the methodological and scientific support of NC. The protocol was revised by NC and J-PS. MG drafted the manuscript, apart from the Statistical Analysis part, which was drafted by MC. J-PS, NC, and CL revised critically the article for important intellectual content. All authors approved the final version of the article.

## Funding

Reimbursement of travel, accommodation, and catering costs for the investigator who collected data was provided by the military hospital Hôpital des Armées Clermont-Tonnerre (Brest, France). Reimbursement of travel, accommodation, and catering costs for the voluntary workers from the medico-psychological emergency cell (CUMP-29 renforcée) was provided by the Centre Hospitalier Régional Universitaire de Brest (France) and Association Brestoise pour la Recherche en Psychiatrie (ABREP). Publishing fees will be funded by Association Cardio-Nautique (Brest, France).

## Conflict of Interest

The authors declare that the research was conducted in the absence of any commercial or financial relationships that could be construed as a potential conflict of interest.
