# Supplementary material for: Impact of a Terrorist Attack on the Mental Health of Directly Exposed French Adolescents: Study Protocol for the First Step of the AVAL Cohort Study
Source: Front Psychiatry. 2019 Oct 25;10:744. doi: 10.3389/fpsyt.2019.00744 (PMC6823664; doi:10.3389/fpsyt.2019.00744)
Supplement: Supplementary file 1 [file Image_1.pdf]

Supplementary Material

1 Supplementary Figures

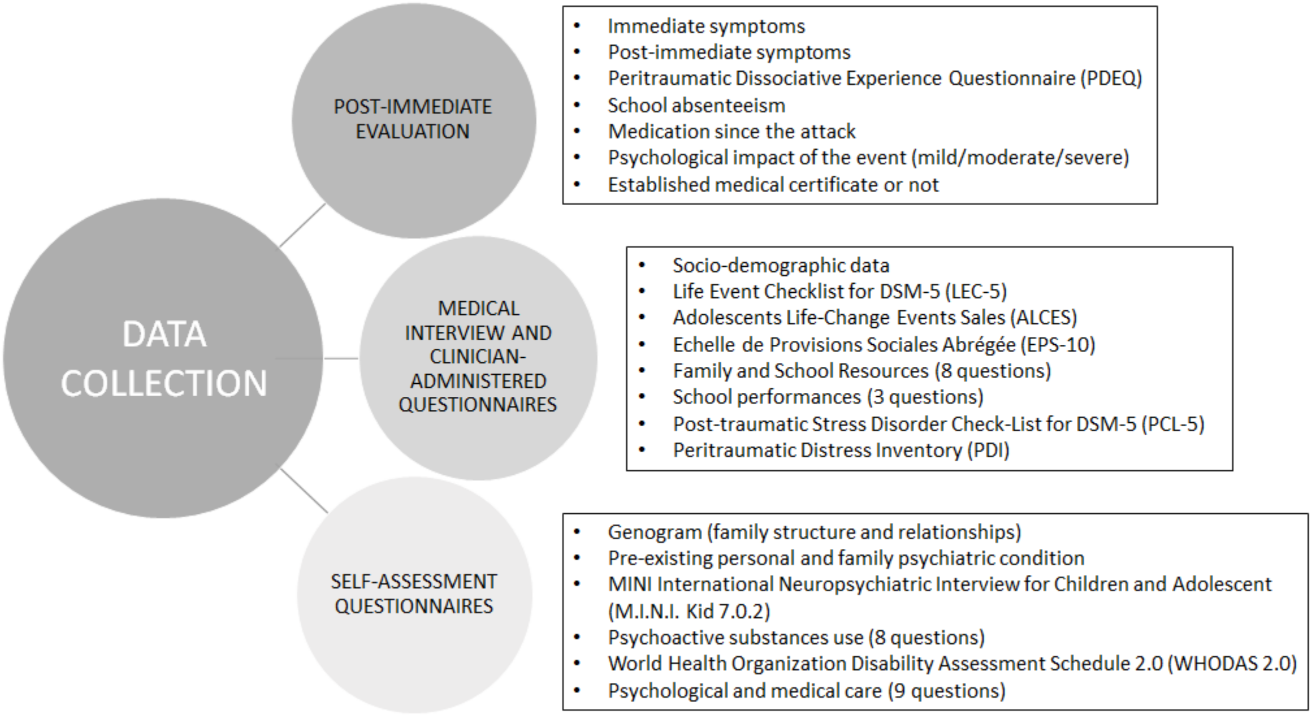

Supplementary Figure 1. Variables assessed in the study
